# ViraLite: an ultracompact HIV viral load self-testing system with internal quality control

**DOI:** 10.1038/s41378-026-01343-9

**Published:** 2026-07-29

**Authors:** Aneesh Kshirsagar, Anthony J. Politza, Tianyi Liu, Md. Ahasan Ahamed, Ming Dong, Muhammad Asad Ullah Khalid, Roland Jones, Uttara Seshu, Kathryn Risher, Casey N. Pinto, Yusheng Zhu, Samir K. Gupta, Weihua Guan

**Affiliations:** 1 Department of Intelligent Systems Engineering, Luddy School of Informatics, Computing, and Engineering, Indiana University, Bloomington, IN 47408 USA; 2 Department of Biomedical Engineering, The Pennsylvania State University, University Park, PA 16802 USA; 3 Department of Electrical Engineering, The Pennsylvania State University, University Park, PA 16802 USA; 4 Departments of Pathology and Laboratory Medicine and Pharmacology, Milton S. Hershey Medical Center and The Pennsylvania State University College of Medicine, Hershey, PA 17033 USA; 5 Department of Public Health Sciences, The Pennsylvania State University College of Medicine, Hershey, PA 17033 USA; 6 The Pennsylvania State University Cancer Institute, Cancer Control Program, Hershey, PA 17033 USA; 7 Division of Infectious Diseases, Indiana University School of Medicine, Indianapolis, IN 46202 USA

**Keywords:** Electrical and electronic engineering, Micro-optics, Biosensors

## Abstract

Effective antiretroviral therapy has transformed HIV into a manageable chronic condition, provided viral suppression is maintained through routine viral load (VL) monitoring. Access to frequent VL testing remains limited, particularly outside centralized clinical settings. Decentralized and at-home HIV VL testing requires low-volume systems designed for patient operation. These systems must distinguish true viral suppression from test failure. Many approaches lack internal process verification, which makes negative results ambiguous. Here, we present ViraLite, an ultracompact, battery-powered HIV VL monitoring system that integrates reverse transcription loop-mediated isothermal amplification (RT-LAMP) with an RNase P internal process control, machine learning–assisted fluorescence analysis for one-pot multiplexing, and smartphone-guided operation. We evaluated ViraLite using 45 clinically archived plasma samples and benchmarked it against reverse transcription quantitative polymerase chain reaction (RT-qPCR). The internal process control identified 17 inconclusive tests that would otherwise be misclassified as negative. Among valid tests, ViraLite achieved 93.3% sensitivity and 100% specificity versus RT-qPCR. ViraLite enables decentralized HIV VL testing with low sample volume and interpretable negative results. This capability can expand monitoring beyond traditional clinic workflows and addresses a key barrier to patient-operated VL testing.

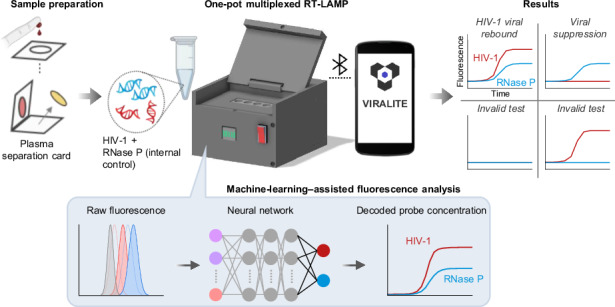

## Introduction

In 2024, of the estimated 40.8 million People Living with HIV (PLHIV) worldwide, approximately 31.6 million (77%) were receiving antiretroviral therapy (ART)^1–3^. Advances in ART have reduced HIV-related mortality by approximately 70% since 2004 and significantly lowered transmission rates^1,4^, transforming HIV into a chronic, manageable condition^5–7^. Yet, in the absence of a curative therapy^8,9^, sustained viral suppression and monitoring remain essential to prevent treatment failure and transmission. Sustained viral suppression below detectable levels reduces transmission risk^10^, but long-term suppression requires periodic assessment of ART. HIV’s high mutation rate^11^ and the emergence of drug-resistant variants increases the risk of treatment failure, and continued transmission, especially as the population of PLHIV grows. Early, accurate, and reliable detection of viral rebound through serial testing is therefore critical for timely treatment adjustment and HIV management^12^.

Because of the stigma associated with HIV, viral load (VL) testing must avoid false-positive results that may cause unnecessary psychological distress, and also maintain sufficient analytical sensitivity to detect low-level viremia reliably^13^. Current clinical practice relies on nucleic acid testing (NAT), typically performed every 3–6 months unless treatment adherence concerns arise^14^. While NATs provide superior sensitivity and specificity compared with antigen- or antibody-based assays^15^, standard workflows require venous blood samples, specialized instrumentation, and multistep sample preparation, with quantitative polymerase chain reaction (qPCR) serving as the gold standard for viral RNA quantification^16–18^. As a result, turnaround times are often several days to weeks, limiting the feasibility of frequent monitoring and potentially delaying clinical decision-making^2,3,12,19–21^.

To improve accessibility, point-of-care (POC) VL platforms such as Roche COBAS AmpliPrep and C6000 analyzer, Cepheid Xpert HIV-1 Viral Load, and Abbott m-PIMA integrate sample preparation, amplification, and detection into automated systems. While these platforms reduce hands-on time, their benefits remain largely confined to healthcare settings. The requirement for relatively large venous blood sample volumes remains a major barrier to translating for at-home or patient-operated use.

Finger-prick blood has been validated for HIV testing, but small sample volumes available (25–100 µL) impose stringent requirements on assay sensitivity and robustness, while reducing tolerance to sample loss. Loop-mediated isothermal amplification^22^ (LAMP) has emerged as an alternative to PCR and has been widely explored for decentralized and portable NAT because of its compatibility with compact instrumentation and simplified workflows^23–25^. LAMP offers high sensitivity, rapid results without thermal cycling^26,27^. Prior studies, including our own, have demonstrated its potential for HIV VL monitoring^23^.

However, challenges remain in applying LAMP to HIV VL monitoring, including susceptibility to non-specific amplification and limited multiplexing capability^28–30^. In parallel, broader nucleic acid amplification literature has highlighted the importance of internal process controls for distinguishing true target-negative results from assay failure or insufficient sample input^31,32^. Conventional single-target LAMP assays lack intrinsic verification of successful sample processing or amplification when viral RNA is not detected, making it difficult to distinguish true viral suppression from assay failure or insufficient sample input. In decentralized, low-volume testing environments, this limitation can lead to misinterpretation of invalid tests as true negatives, potentially delaying identification of treatment failure. Although prior HIV RT-LAMP studies have demonstrated single-target detection^33^, portable VL testing^23^, and in some cases inclusion of an internal control^34^, these approaches have not established a compact one-pot multiplex workflow that combines HIV detection with integrated process control for reliable sample processing and test evaluability in decentralized use. Accordingly, there remains an unmet need for compact HIV VL testing systems that integrate internal process controls to enable reliable interpretation of results in decentralized, low-volume, patient-operated settings.

In this work, we present ViraLite, an ultracompact HIV VL monitoring system that addresses these challenges through multiplexed reverse-transcription LAMP (RT-LAMP) with internal quality control, aimed toward decentralized use. The system enables low-volume HIV VL testing from plasma samples by combining probe-based multiplex RT-LAMP, semi-automated sample processing, and a compact, battery-powered analyzer. To enable true one-pot multiplexed detection of HIV RNA and RNase P (internal process control) within a single reaction, we implement a machine-learning–assisted fluorescence analysis framework that reliably resolves overlapping signals from multiple fluorophores. In addition, a smartphone-guided interface provides step-by-step workflow guidance and real-time feedback, supporting consistent execution of the testing procedure in patient-operated settings. We evaluate the system using clinically archived plasma samples and benchmark its performance against RT-qPCR, demonstrating the importance of internal process control and machine-learning-enabled multiplexing for reliable at-home HIV VL monitoring.

## Results

### ViraLite workflow

**Fig. 1:**
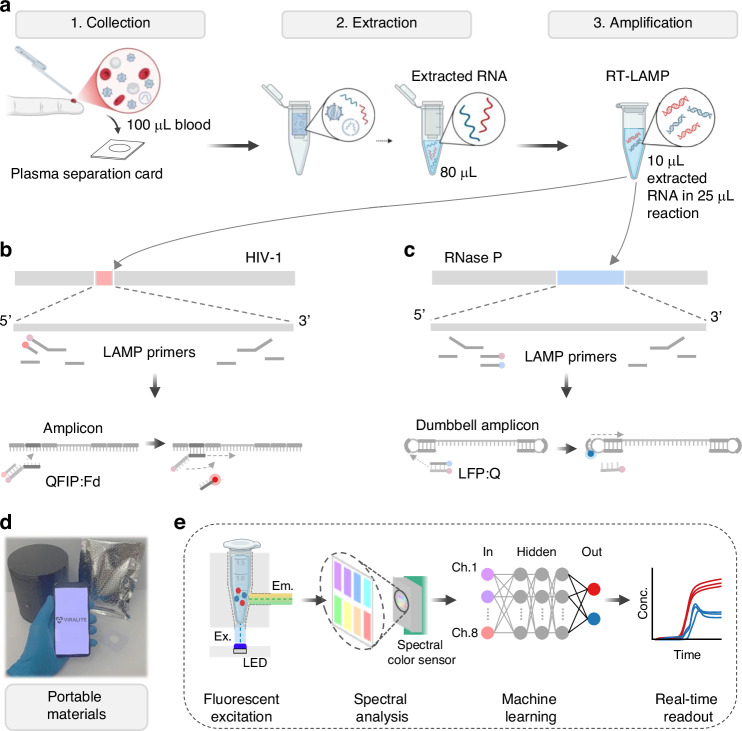
Demonstration of portable VL monitoring for self-management using ViraLite protocols. **a** Samples are first gathered using a plasma separation card that filters finger-prick blood and traps plasma into an absorption layer. Next, that layer is transferred to lysis buffer, and RNA extraction is conducted using a custom-developed portable centrifuge to process QIAamp viral RNA extractions. Lastly, the isolated RNA is added to our multiplex RT-LAMP assay for real-time analysis using our portable analyzer. **b** The HIV-1 components of the multiplex assay target the reverse-transcriptase sub-region of the viral genome. A designed probe, using the FIP primer attached with a quencher, incorporates into the reaction as amplicons are generated and causes a change in fluorescence as a complementary fluorophore is released into the bulk assay. **c** The RNase P components of the multiplex assay target the mRNA strand ‘RPP subunit p20’. Another designed probe, using the LF primer attached with a fluorophore, incorporates into the reaction as dumbbell amplicons are generated and causes a change in fluorescence as a complementary quencher is released into the bulk assay. **d** The entire test can be packaged into a vacuum-sealed pouch; extractions are verified for long-term storage without cold-chain dependency. The RT-LAMP assay still necessitates refrigeration. **e** Detecting our multiplex assay is accomplished through a fine integration of hardware and software. The handheld device reads excited photons (480 nm Blue LED) from the bulk assay using a multi-spectra color sensor. The multi-spectral color sensor outputs 8 channels of usable RFU that can then be converted to dye/fluorophore concentration using a trained neural network (NN). Concentrations are then displayed to users via a smartphone app or a provided summary tab

To enable at-home VL monitoring via nucleic acid testing, we designed and validated a portable sample-to-answer platform (Fig. 1a). The system integrates our previously validated plasma separation membrane for finger-prick blood collection and plasma separation (step i)^23^, a light-weight centrifuge for portable RNA extraction (step ii)^35^, and a newly developed multiplex RT-LAMP assay on a portable analyzer (step iii). A smartphone app guides users through sample collection, RNA extraction, and amplification (Supplementary Video S1). The total turnaround time of the ViraLite workflow, including sample collection, RNA extraction, amplification, and result reporting, was approximately 75 min. Finger-prick blood collection was designed to obtain approximately 100–140 µL of whole blood, with the marked circular guide indicating the 100 µL target fill volume. RNA extraction yielded 80 µL of eluate, of which 10 µL was used as input into the 25 µL multiplexed RT-LAMP reaction. The RT-LAMP assay employs custom-designed probes to improve target specificity in a multiplexed format (Fig. 1b, c). Detection is performed on ViraLite, a compact fluorescence analyzer (85 × 80 × 55 mm; 245 g) that uses a machine-learning pipeline to analyze multispectral sensor data and enable one-pot multiplexing (Fig. 1d, e). Amplification is performed directly on ViraLite, providing real-time results to the user via a Bluetooth-connected app.

### HIV RT-LAMP assay with internal quality control

To incorporate internal quality control, we developed two probe-based RT-LAMP assays, with assay mechanisms described in the *Supplementary Information.* Each assay was first evaluated in single-plex format before one-pot multiplexing. The HIV RT-LAMP assay targets the reverse transcriptase region of the HIV genome and achieved a limit of detection (LOD) of 13 copies at the 95% confidence level (Supplementary Fig. S2a). The RNase P RT-LAMP assay targets the RPP subunit p20 RNA and demonstrated a limit of detection of 4 copies at the 95% confidence level (Supplementary Fig. S2b). Both assays showed strong agreement between benchtop and portable platforms (Supplementary Fig. S3a, b), supporting their suitability for multiplexed deployment.

**Fig. 2:**
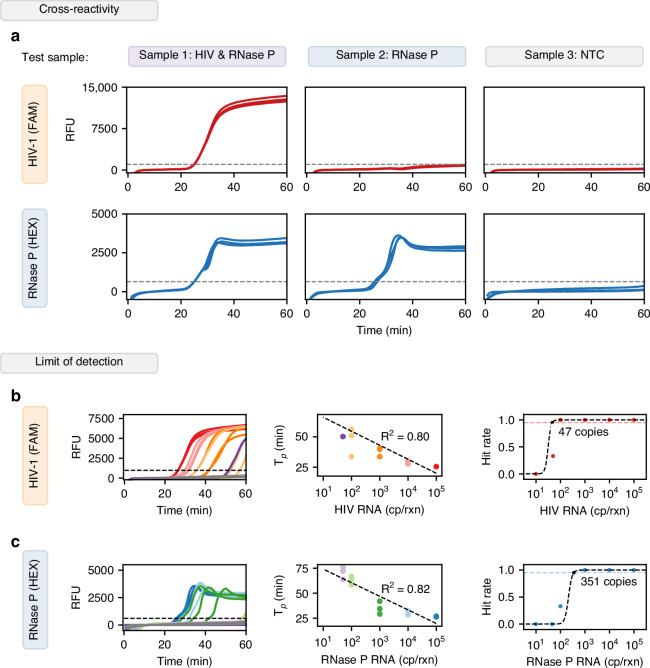
Validation of multiplex assay using cross-reactivity and limit of detection. **a** The multiplex assay used FAM and HEX fluorophores to distinguish HIV-1 and RNase P, respectively. When both targets are present in the sample (left), exponential amplification occurs for both FAM and HEX. For samples containing only RNase P (middle), amplification is seen only in the HEX channel. No-Template Control (NTC, right) shows no amplification for either FAM or HEX (HIV-1 or RNase P). **b** Amplification curves using the multiplex assay to detect HIV-1 show positive detection from 10^5^ down to 47 cp/reaction (left). The multiplex assay shows a linear relationship between HIV-1 RNA concentration and Time to Positive (T_p_) (middle). The hit rate analysis for HIV-1 demonstrated a LOD of 47 copies using the multiplex assay (right), established using a 95% confidence level. **c** Amplification curves using the multiplex assay to detect RNase P show positive detection from 10^5^ down to 351 cp/reaction (left). The multiplex assay shows a linear relationship between RNase P RNA concentration and T_p_ (middle). The hit rate analysis for RNase P demonstrated a LOD of 351 copies using the multiplex assay (right), established using a 95% confidence level

Assay cross-reactivity was evaluated by testing three sample variations: HIV and RNase P positive (simulating viral rebound), RNase P positive (simulating a patient sample without viral rebound), and a No-Template Control (NTC, simulating an invalid test). Each variation was tested in triplicate using the one-pot multiplexed assay on a benchtop thermal cycler (BioRad CFX96). Only the expected target-channel combinations produced amplification (Fig. 2a): sample 1 showed detection of both HIV and RNase P; sample 2 showed detection of only RNase P; and sample 3 showed no detection of either target, indicating high specificity and absence of primer interactions. Identical results were obtained using the ViraLite analyzer (Supplementary Fig. S4), demonstrating robust multiplex performance across platforms in clinically relevant scenarios.

The LOD of the multiplexed RT-LAMP assay was evaluated by testing HIV-1 samples ranging from 10^5^ to 10 copies per reaction. As shown in Fig. 2b, the multiplexed assay performed similarly to the single-plex assay, showing a good linearity (R^2^ = 0.80) over the range of concentrations detected. Following a hit curve analysis, where the hit rate is defined as the number of positive tests divided by the total number of tests performed at the same concentration, the assay showed an LOD of 47 copies, suggesting the potential for semi-quantitative detection of HIV and for accurate analysis of 100 µl samples containing concentrations as low as 1 cp/µl (1000 cp/mL). Testing RNase P RNA over an identical concentration range (10⁵ to 10 copies per reaction) showed that multiplexed assay performance was comparable to its single-plex counterpart, with good linearity (R² = 0.82) and a limit of detection of 351 copies (Fig. 2c). Although this limit was higher than anticipated, the assay is still expected to reliably detect RNase P in plasma samples (~12 copies/µL) (Supplementary Fig. S5).

### Validation of multiplex detection

**Fig. 3:**
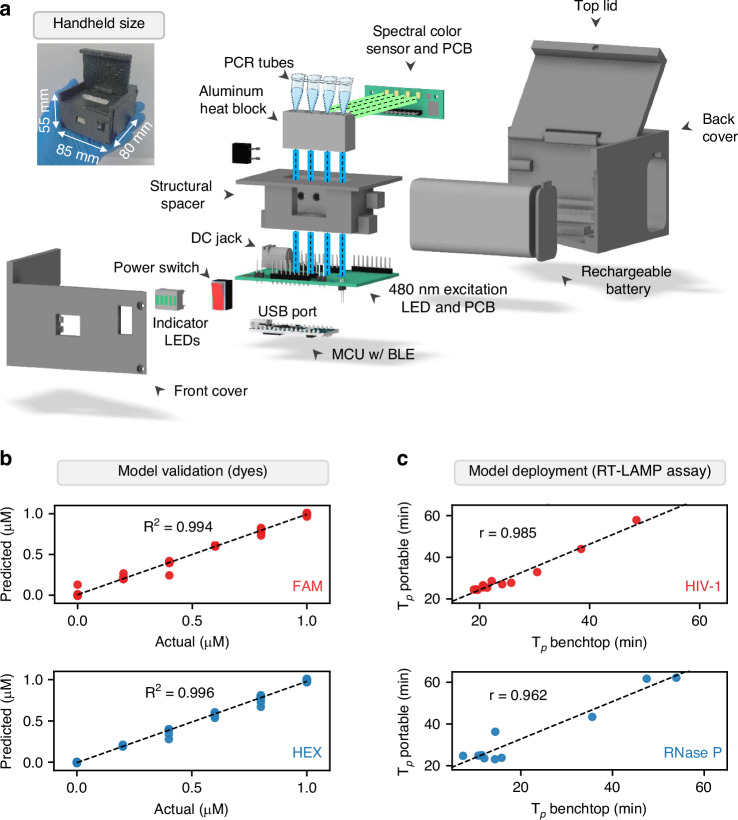
Validation of the hardware and software of the ViraLite analyzer. **a** The ViraLite analyzer is composed of several electrical components housed within a custom, 3D printed enclosure. The 480 nm excitation LEDs are located on the horizontal mainboard, underneath the structural spacer and heat block. Light from these LEDs (shown in blue) will travel vertically upward into the heat-block, where the PCR tubes for the assay are located. Once excited, the assay will emit photons (light path shown in green), which are detected by the spectral color sensor located orthogonal to the excitation LED. A BLE-enabled MCU is located on the bottom of the mainboard PCB, connects the analyzer’s components, monitors internal feedback, and transmits data. **b** The analyzer and machine learning software (neural network) showed excellent performance in detecting increasing dye concentrations (FAM and HEX). **c** The analyzer showed very strong agreement with the benchtop thermal cycler for analyzing both targets (HIV-1 and RNase P) in multiplex format over a range of concentrations (10^5^ to 10 copies)

To enable a compact, battery-powered diagnostic device capable of multiplexed detection, we trained and integrated a neural network (NN) that converts 8-channel fluorescence measurements into FAM and HEX concentrations, building on prior work demonstrating superior performance for resolving overlapping emission spectra^36^. In the present configuration, only the 480 nm excitation source was used. Under this excitation condition, FAM and HEX generated overlapping but distinguishable responses across the 8-channel spectral sensor (Supplementary Fig. S6). Because these responses overlap across channels, interpretation based on any single channel is insufficient; therefore, the full 8-channel fluorescence signature was used as input to the NN to predict fluorophore-specific concentrations. These NN-predicted FAM and HEX concentrations were then plotted over time to generate the amplification curves used for diagnostic interpretation. This approach enables a lightweight and simplified optical design while maintaining high analytical accuracy (Fig. 3a). Model performance was evaluated using synthetic dye mixtures and multiplexed real-time RT-LAMP assays. In total, 36 combinations of FAM and HEX across six concentrations (0, 0.2, 0.4, 0.6, 0.8, 1 µM) were prepared, with 10 measurements per combination, replicated four times, and split 80/20% for training and testing. Figure 3b compares NN-predicted dye concentrations with expected values, demonstrating strong linearity for both FAM (R^2^ = 0.994) and HEX (R^2^ = 0.996), consistent with our prior work^18,37^. Prediction errors were low, with MAE and RMSE of 0.013 and 0.029 µM for FAM and 0.014 and 0.029 µM for HEX, corresponding to approximately 1–3% of the fluorescence range. Residuals were centered near zero with a narrow distribution, while Bland–Altman analysis showed minimal systematic bias and consistent agreement across the fluorescence range (Supplementary Fig. S7), supporting reliable fluorescence quantification for multiplexed real-time assays. Additional details of the model architecture, training procedure, and evaluation are provided in the Supplementary Information. To verify its deployment for the real-time multiplexed assay, HIV-1 samples (containing RNase P) were tested on the analyzer in a concentration range of 10^5^ to 10 copies/rxn, with parallel testing performed on a benchtop thermal cycler (BioRad CFX96). Figure 3c compares the time-to-positive (T_*p*_) values measured on both platforms. The portable ViraLite analyzer showed strong agreement with the benchtop system for both HIV (*r* = 0.985) and RNase P (*r* = 0.962). The strong correlation and >96% agreement indicate that the integrated NN model is accurate and reproducible, supporting the suitability of ViraLite for portable multiplexed assay analysis.

### Deploying ViraLite for qualitative testing

**Fig. 4:**
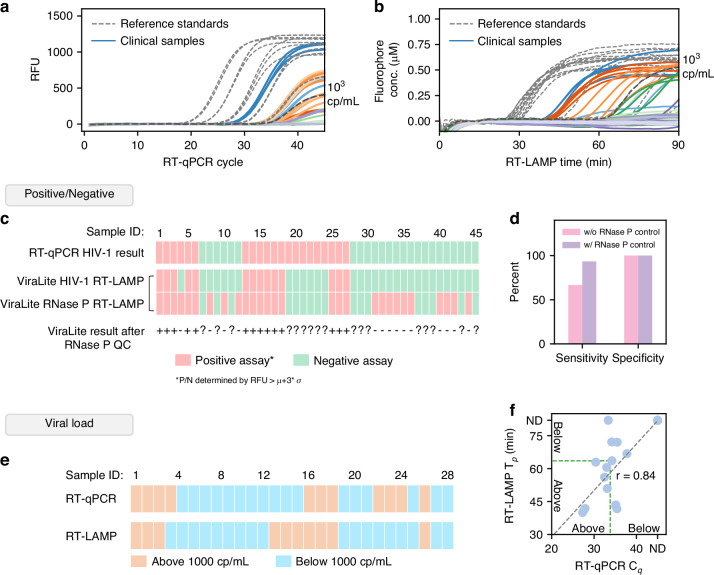
Clinical demonstration of ViraLite using sensitivity, specificity, and clinically relevant viral load classification based on the 1000 cp/mL threshold. **a** Amplification curves from 45 clinical samples using RT-qPCR as a benchmark. A 1000 cp/mL marker is designated as a dark gray dashed line. **b** Amplification curves from 45 clinical samples, analyzed using our multiplex RT-LAMP assay on our portable ViraLite analyzer (data smoothing applied with Python). Colored traces represent clinical sample amplification curves, with replicate curves from the same sample grouped together by color for visual separation. Dashed light gray traces represent reference standards. A 1000 cp/mL reference standard is represented by a dark gray long-dashed trace. **c** Sample-by-sample endpoint comparison for all 45 clinical samples between the reference RT-qPCR HIV-1 result and the corresponding ViraLite HIV-1 result after RNase P quality control. Positive results are shown in red and negative results are shown in green. For ViraLite, positive/negative calling was established using an RFU threshold of µ + 3σ. **d** Clinical sensitivity and specificity of our multiplex RT-LAMP assay with and without the internal quality control to eliminate invalid samples. **e** Viral load classification of RT-qPCR vs our multiplex RT-LAMP assay for 28 clinical samples, in which RNase P was detected by multiplexed RT-LAMP. The typical clinical threshold of 1000 cp/mL was used to define Above (>1000 cp/mL) and Below (<1000 cp/mL) classifications. **f** Scatter plot of RT-LAMP T_*p*_ versus RT-qPCR C_*q*_ values for RNase P–valid samples (*n* = 28). Dashed lines indicate assay-specific thresholds used to classify samples as above or below 1000 copies/mL. Concordant and discordant classifications are visualized by quadrant, with most discrepancies occurring near the decision boundaries

To validate the analyzer’s qualitative performance, its clinical sensitivity and specificity were evaluated using 45 archived plasma samples. Each sample was split into two 50 µl aliquots. The first was processed and analyzed using a standard laboratory workflow that included a benchtop centrifuge (Eppendorf 5425) and RT-qPCR instrument (BioRad CFX96). The amplification curves and corresponding RNA concentrations for these samples are shown in Fig. 4a. The second aliquot was processed and analyzed using our portable extraction protocol and multiplex RT-LAMP ViraLite analyzer, with amplification curves shown in Fig. 4b.

For qualitative analysis, endpoint results are summarized in Fig. 4c with real-time quantitative values provided in Supplementary Table S7. The amplification curves used for this analysis were not derived from a single raw sensor channel; rather, at each time point, fluorescence recorded across all 8 spectral channels was processed by the NN to generate predicted FAM and HEX concentration trajectories, which were then used for threshold-based positive/negative calling. RT-qPCR identified 21 positive and 24 negative samples. Considering only the HIV detection channel on ViraLite using RT-LAMP, 14 positive and 31 negative samples were detected, corresponding to a modest sensitivity of 66.7% (14/21; 95% CI: 43.0 to 85.4%) and a high specificity of 100% (24/24; 95% CI: 85.8 to 100%). Leveraging RNase P internal quality control enabled the identification of 17 invalid or inconclusive tests, defined as the absence of RNase P amplification, regardless of HIV RT-qPCR result. In the refined dataset comprising 15 PCR-positive and 13 PCR-negative samples, ViraLite’s sensitivity increased to 93.3% (14/15; 95% CI: 68.1 to 99.8%), while specificity remained 100% (13/13; 95% CI: 75.3 to 100%) (Fig. 4d). These RNase P-negative results likely reflect the limited and variable amount of host nucleic acid present in archived plasma, together with RNA extraction and isolation-related losses and possible matrix-associated inhibition affecting RT-LAMP. Together, these results demonstrate the strong qualitative performance of ViraLite for clinical sample classification.

### Deploying ViraLite for clinically relevant viral load classification

Beyond qualitative HIV detection, we next evaluated whether ViraLite could support classification of valid samples relative to the clinically relevant VL threshold of 1000 cp/mL. For this analysis, each clinical sample deemed valid by the RT-LAMP assay based on RNase P detection was classified as ‘above’ or ‘below’ 1000 cp/mL using RT-qPCR C_*q*_ and RT-LAMP T_*p*_ values. A VL below 1000 cp/mL is a clinically established threshold associated with viral suppression and markedly reduced transmission risk^4,38–40^. Assay-specific C_*q*_ and T_*p*_ cutoffs were defined using five reference concentrations (dashed lines in Fig. 4a, b), which also confirmed assay linearity. Classification results are summarized in Fig. 4e. ViraLite analyzer running multiplexed RT-LAMP classified 10 samples as above and 18 as below the threshold, whereas RT-qPCR classified 11 as above and 17 as below. Overall agreement between the two methods was 75.0%, with a positive percent agreement (PPA) of 63.6% and a negative percent agreement (NPA) of 82.4%. Figure 4f compares ViraLite T_*p*_ with RT-qPCR C_*q*_ values. Dashed lines denote assay-specific thresholds corresponding to a VL of 1000 copies/mL, which divide samples into above- and below-threshold categories for each method. Samples cluster predominantly within concordant quadrants, while discordant cases are observed near the decision boundaries, consistent with the limited quantitative resolution of RT-LAMP relative to RT-qPCR.

### Surveying ViraLite perception among patients in HIV clinics

**Fig. 5:**
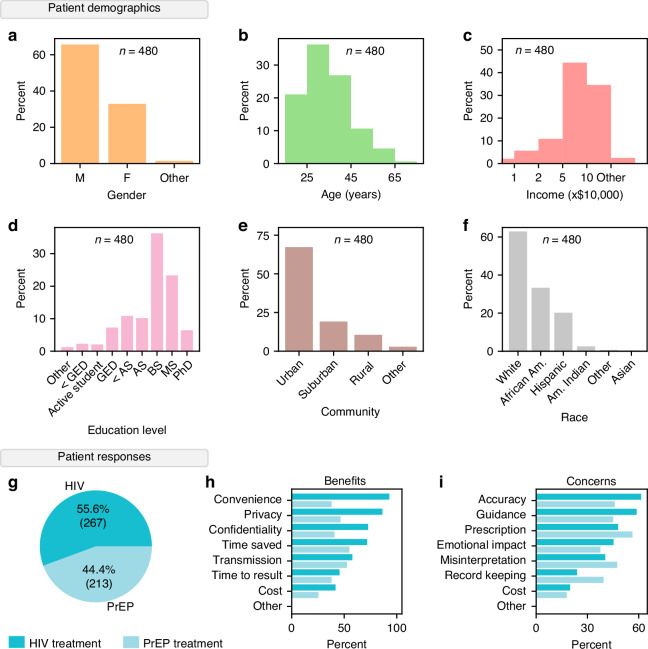
Evaluation of survey participant feedback toward ViraLite improvements. The distribution of participants (*n* = 480) for each demographic: **a** Gender, **b** Age, **c** Income, **d** Highest Education level, **e** Community, and **f** Race. **g** Patient responses indicated a 55.6% (HIV) to 44.4% (PrEP) separation between current treatments. **h** Percent indication of the perceived benefits of our system from patients on either treatment (HIV – dark, PrEP – light). The highest percentages of marked benefits were Convenience, Privacy, and Confidentiality (HIV treatment). **i** Percent indication of the potential concerns of our system from patients on either treatment (HIV – dark, PrEP – light). The highest percentages of marked concerns were Accuracy, Guidance, and Prescription [management] (HIV treatment)

To assess potential acceptance and usability, 480 patients receiving HIV or pre-exposure prophylaxis (PrEP) treatment were surveyed. Voluntary participants at several HIV clinics were asked to watch a short instructional video demonstrating the operation of ViraLite (Supplementary Video S2), followed by a series of demographic and response questions. Patient demographics, summarized by gender, age, income, education level, urban or rural status, and race, are presented in Fig. 5a–f, and align with the 2023 U.S. Statistics for HIV^41^, indicating a broad and representative survey population (see *Supplementary Information*). Survey responses were further categorized by treatment plan, including HIV antiretroviral therapy, or PrEP (Fig. 5g). 55.6% of patients were receiving HIV treatment, while 44.4% were on PrEP.

Among participants receiving HIV treatment, more than half indicated Convenience, Privacy, Confidentiality, and Time Saved as their most perceived benefits (Fig. 5h). Participants on PrEP prioritized benefits related to Time Saved, decreased risk of Transmission, and Privacy. Fewer than half of all participants were interested in the benefits of Time to Results and Cost. In contrast, participants receiving HIV treatment expressed the greatest concern regarding Accuracy, Guidance, and Prescription (Fig. 5i). Participants on PrEP reported the greatest concern for Prescription [management], Misinterpretation [of results], Accuracy, and Guidance. Less than half of all participants expressed concern about Emotional Impact [of tests/results], Record Keeping, or Cost. These findings highlight areas for continued refinement as ViraLite is further developed. Overall, survey feedback indicates strong potential for a positive patient impact, while informing future improvements as the technology is translated into patients’ hands.

## Discussion

Portable, sensitive, and specific assays are needed to enable VL monitoring closer to the patient. ViraLite’s probe-based multiplexed RT-LAMP assay supports low-volume sampling while maintaining high sensitivity and specificity. Incorporation of RNase P amplification as an internal control improves result interpretability and underscores the importance of test evaluability in decentralized molecular diagnostics; rather than enhancing the intrinsic analytical sensitivity of RT-LAMP chemistry, RNase P screening identifies invalid or inconclusive reactions. During archived clinical sample testing, a substantial number of samples that would otherwise have been classified as HIV-negative were identified as invalid due to the absence of RNase P amplification. This reclassification accounted for the observed increase in sensitivity and demonstrates how implementing process control improves test reliability in low-volume conditions. However, the raw sensitivity of the full workflow before exclusion of invalid or inconclusive tests was 66.7%. This lower value reflects the combined influence of sample preparation, assay execution, and test evaluability in the current low-volume workflow for decentralized use. After RNase P-based quality control was applied, sensitivity increased to 93.3% in the evaluable subset. The RNase P-negative inconclusive results most likely reflect the limited and variable abundance of host nucleic acid in archived plasma, which is a relatively cell-poor matrix, together with sample-preparation-related losses and possible matrix-associated RT-LAMP inhibition. Together, these findings highlight both the value of internal quality control and the need for further improvement in end-to-end workflow robustness.

The integration of RT-LAMP–based HIV VL monitoring with machine learning and a battery-powered analyzer bridges the gap between clinic-based testing and patient-centered monitoring. Machine learning enables ViraLite to implement HIV and RNase P detection in a single reaction by resolving overlapping fluorescence signals required for reliable one-pot multiplexing. This is achieved using simple, off-the-shelf components, eliminating complex optical filters or lens-based imaging optics while maintaining low prediction error relative to decision thresholds, as supported by residual and agreement analyses. This approach enables robust fluorescence interpretation in a compact form factor suitable for portable and potentially at-home use. Agreement between portable and benchtop measurements indicates that computational signal separation can reliably support multiplexed RT-LAMP readout.

RT-LAMP VL classification was compared with RT-qPCR for archived clinical plasma samples using a clinically relevant VL threshold of 1000 copies/mL. This analysis is best interpreted as clinically relevant threshold-based VL classification rather than absolute quantification across multiple VL tiers. Moderate agreement was observed, with discordant classifications primarily near the decision threshold, reflecting inherent differences in amplification mechanisms and signal generation dynamics. RT-qPCR C_*q*_ values reflect exponential amplification under thermal cycling, whereas RT-LAMP T_*p*_ depends on isothermal kinetics and cumulative fluorescence accumulation. The modest linearity observed for the multiplexed RT-LAMP assay is also consistent with limited quantitative resolution relative to fully quantitative methods such as RT-qPCR. Accordingly, the multiplexed RT-LAMP format is better interpreted as supporting threshold-based VL classification and internal quality control rather than precise absolute quantification across a broad dynamic range. The clustering of discordant samples near the classification boundary indicates bounded and predictable uncertainty, supporting the use of RT-LAMP for treatment-relevant VL categorization.

From a translational perspective, the current laboratory-phase ViraLite analyzer has an approximate cost of $64 and a research-phase reagent cost of approximately $6 per test, both of which are expected to decrease with scale-up. It currently uses a 16.28 Wh lithium-ion battery and consumes approximately 3 Wh per test, enabling up to 5 battery-powered tests while also supporting charging and plugged-in use via a standard wall socket. The extraction reagents are room-temperature stable in vacuum-sealed packaging, whereas the current RT-LAMP assay requires refrigerated storage. In our earlier work, we demonstrated a singleplex lyophilized HIV RT-LAMP assay^23^, and future efforts will focus on extending and optimizing this approach for the present multiplex assay format. For a broader deployment context, a comparison of ViraLite with the Cepheid Xpert HIV-1 VL platform is provided in Supplementary Table S8; this comparison is intended as a general workflow and implementation reference rather than a direct head-to-head performance evaluation.

A user survey assessed perceptions of at-home HIV VL monitoring and identified anticipated benefits and concerns related to ViraLite. Survey responses indicated that the participant population was representative of the intended end users for at-home HIV VL monitoring and that extending VL monitoring beyond clinic settings was perceived as useful, particularly with respect to convenience and privacy. Participants also highlighted concerns related to accuracy, result interpretation, prescription management, and clinical guidance. These concerns suggest several implementation priorities for future versions of ViraLite. Confidence in accuracy can be strengthened through continued assay optimization, retention of internal process controls such as RNase P, and clearer identification of invalid or inconclusive runs so that suboptimal tests are not misread as true negatives. Concerns related to result interpretation could be addressed through an intelligent smartphone-based interpretation layer that converts assay outputs into plain-language categories and recommended next steps. Workflow instruction-related concerns could be addressed through an improved guided smartphone interface with animations for stepwise instructions and error-prevention prompts during sample collection and test execution. Finally, concerns related to prescription management and clinical guidance could be addressed through a secure clinical result docking pathway that supports healthcare provider review, longitudinal monitoring, and follow-up when results indicate potential treatment failure or the need for repeat testing. Although the present survey provided initial insight into anticipated usability and patient acceptance, formal evaluation of operational consistency across users with different skill levels, including non-professional patients and medical staff, will be an important next step in the translational development of ViraLite.

This study has some limitations. The clinical cohort size was modest, which limits statistical power and results in relatively wide confidence intervals around the reported sensitivity and specificity estimates. Larger validation studies, including fresh clinical specimens and broader patient cohorts, will be important for further translational evaluation of ViraLite. Samples classified as negative corresponded to undetectable VLs by RT-qPCR rather than to confirmed HIV-negative specimens; therefore, reported specificity reflects agreement in identifying suppressed or undetectable VLs rather than discrimination between infected and uninfected individuals. Sample preparation remains a key challenge for compact, patient-operated devices, as integrating nucleic acid extraction and testing into a low-cost automated format introduces complexity and potential reliability issues, particularly due to carryover inhibitors. The higher HIV and RNase P limits of detection observed in the multiplexed assay relative to their respective singleplex formats reflect a trade-off inherent to one-pot multiplexing, in which multiple primer and probe sets must operate under shared reaction conditions rather than conditions optimized for a single target. In the present assay, primer concentrations were adjusted to a 3:2 HIV-to-RNase P ratio to preserve strong HIV detection performance while retaining RNase P as an internal quality control target. Consequently, some reduction in analytical sensitivity is expected in the multiplexed format. For RNase P, the resulting multiplexed LOD was still expected to support detection at the level required for sample evaluability in plasma. Further optimization of primer ratios and multiplex reaction conditions may reduce these trade-offs in future iterations, and evaluation of RNase P variability across broader clinical cohorts remains an important direction for future study. Inter-batch reproducibility and environmental robustness, including performance under different temperature and humidity conditions, were not systematically evaluated in the present study. Because ViraLite is intended for use in primary care and home environments rather than uncontrolled field settings, the present study focused on analytical and clinical performance under the intended use context. These parameters remain important for clinical translation and will require dedicated assessment in future studies. In addition, formal real-world usability metrics, such as task completion, user error rates, and operational consistency across users with different skill levels, were not measured here and will require dedicated future study.

In conclusion, ViraLite demonstrates a sensitive, portable approach to self-directed HIV VL monitoring enabled by multiplex RT-LAMP, machine-learning–based fluorescence analysis, and semi-automated, battery-powered electronics. The system integrates sample preparation, multiplex detection, and smartphone-guided operation into an accessible testing platform. Evaluation using archived clinical samples demonstrates real-world feasibility by pairing reliable clinical data with portable hardware. By lowering barriers to frequent VL assessment, ViraLite has the potential to increase monitoring frequency, support improved ART management, reduce transmission risk, and enable more patient-centered HIV care.

## Materials and methods

### RT-LAMP assay

Reactions for HIV-1 detection were performed in a 25 µL volume using an optimized buffer and primer composition (Supplementary Table S1). Amplification was carried out at 60 °C for 60 min. For multiplexed assays, primer concentrations were adjusted to a 3:2 HIV to RNase P ratio to support internal quality control. Primer and probe sequences are listed in Supplementary Table S2.

### RT-qPCR assay

The HIV RT-qPCR assay was performed as previously validated by Palmer et al^42^. Reactions were carried out in a 25 µL volume using a one-step RT-qPCR master mix with extracted RNA as input, targeting a 79-bp amplicon (Supplementary Table S3). Amplification and fluorescence detection were performed on a Bio-Rad CFX96 thermal cycler using standard cycling conditions (Supplementary Table S4). Primer and probe sequences are listed in Supplementary Table S5.

### ViraLite instrumentation

The four-chamber ViraLite analyzer was designed using PTC Creo and fabricated via 3D printing (MakerBot Method X). Custom printed circuit boards (PCBs) were designed in Autodesk Eagle and manufactured by OSH Park, with electronic components assembled in-house. The smartphone app, with user guidance and a graphical user interface (GUI), was developed using MIT App Inventor. The app facilitates control and communication with the device via Bluetooth (Supplementary Fig. S1a, b). Additional information is provided in the Supplementary Information, including a Bill of Materials for the analyzer in Supplementary Table S6.

### Sample preparation with a portable device

A custom portable centrifuge was used to extract viral RNA from a 50–100 µl sample, using the QIAamp Viral RNA Mini Kit (Qiagen), following our previously established protocol^35^. Our smartphone app guides the user through the extraction process (refer to Supplementary Video S1).

### Sample preparation with a benchtop device

Benchtop RNA extractions were performed on 100–140 µL samples using the QIAamp Viral RNA Mini Kit (Qiagen) on an Eppendorf 5425 centrifuge, following the manufacturer’s protocol.

### Clinical samples

Plasma samples were collected from HIV patients treated at the Penn State Hershey Medical Center following a controlled standard operating procedure. Institutional Review Board (IRB) approval was provided by The Pennsylvania State University (STUDY00015905). The samples were de-identified and randomly selected from remnant plasma batches. No identifiable data was recorded or transferred between study locations. For each test, 50 µl of clinical plasma sample was used.

### Survey recruitment

Survey participants were recruited online and at the Penn State Hershey Medical Center, the UPMC infectious disease center, the Penn Health Lancaster General Health Comprehensive Care Center, the Seattle King County HIV/STI clinics, and the Washington University HIV/STI clinics. A total of 1200 surveys were collected. Surveys from participants who watched more than 30 s of the video were retained, resulting in 480 responses for analysis. Institutional Review Board (IRB) approval was obtained from The Pennsylvania State University (STUDY00024241). Participants were compensated with a $10 Amazon gift card for the successful completion of the survey.

### Statistical analysis

All data processing and figure generation were completed using Python. Data is displayed as the mean of triplicates plus or minus three standard deviations, unless otherwise noted. Positive samples are classified using Time to Positive (T_*p*_) or quantitative cycle (C_*q*_) when RFU exceeds a threshold of μ + 3σ. LOD was calculated with 95% confidence using a logistic function fit to the hit rate for each assay. Correlation and linearity were computed using SciPy and least squares regression.

## Supplementary information

Supplementary Information file

Supplementary Video S1

Supplementary Video S2
