# Supplementary material for: ViraLite: an ultracompact HIV viral load self-testing system with internal quality control
Source: Microsyst Nanoeng. 2026 Jul 29;12:282. doi: 10.1038/s41378-026-01343-9 (PMC13415889; doi:10.1038/s41378-026-01343-9)
Supplement: Supplementary file 1 — Supplementary Information file [file 41378_2026_1343_MOESM1_ESM.docx]

**ViraLite: An Ultracompact HIV Viral Load Self-Testing System with Internal Quality Control**

Aneesh Kshirsagar^1†^, Anthony J. Politza^2†^, Tianyi Liu^3^, Md. Ahasan Ahamed^1,3^, Ming Dong^3^, Muhammad Asad Ullah Khalid^1^, Roland Jones^4^, Uttara Seshu^5^, Kathryn Risher^5^, Casey N. Pinto^5,6^, Yusheng Zhu^4^, Samir K. Gupta^7^ and Weihua Guan^1*^

1. Department of Intelligent Systems Engineering, Luddy School of Informatics, Computing, and Engineering, Indiana University, Bloomington, IN 47408, United States
2. Department of Biomedical Engineering, The Pennsylvania State University, University Park, PA 16802, United States
3. Department of Electrical Engineering, The Pennsylvania State University, University Park, PA 16802, United States
4. Departments of Pathology and Laboratory Medicine and Pharmacology, Milton S. Hershey Medical Center and The Pennsylvania State University College of Medicine, Hershey, PA 17033, United States
5. Department of Public Health Sciences, The Pennsylvania State University College of Medicine, Hershey, PA 17033, United States
6. The Pennsylvania State University Cancer Institute, Cancer Control Program, Hershey, PA 17033, United States
7. Division of Infectious Diseases, Indiana University School of Medicine, Indianapolis, IN 46202, United States

* Corresponding author’s email address: guanw@iu.edu

^†^ Drs. Kshirsagar and Politza contributed equally to this work and should be considered co-first authors.

Contents

[Supplementary Information 3](#_Toc192839305)

[Supplementary Figures 6](#_Toc192839306)

[Supplementary Tables 12](#_Toc192839307)

[Supplementary Videos 15](#_Toc192839308)

# Supplementary information

## RT-LAMP probe design

Earlier studies into multiplexed LAMP have demonstrated probe-based detection using labeled primers^1–3^. Detection of Amplification by Release of Quenching (DARQ) supplements the standard LAMP primers with a duplex probe^4,5^. In this method, the duplex consists of a quencher attached forward inner primer (QFIP) annealed to a fluorophore labeled F1 region (Fd)^4^. Another method of probe-based LAMP, 5p-OSD, also supplements the standard LAMP assay with a duplex probe^6,7^. The difference between DARQ and 5p-OSD is apparent in the utilization of one of the standard loop primers (LP) as one-half of the added duplex^7^. In this manner, 5p-OSD is designed with a 5’-fluorophore and annealed to a complementary strand containing a 3’ quencher (Qd). Our design uses both DARQ (HIV-1) and 5p-OSD (RNase P) to achieve a multiplex LAMP assay for HIV-1 and RNase P.

## ViraLite analyzer design

The ViraLite analyzer architecture builds upon prior work^8^ demonstrating that single-channel fluorescence monitoring limits multiplexing capability (Supplementary Figure S5). To address this limitation, the system employs a multi-spectral optical sensing approach, enabling analysis of broad-spectrum assay fluorescence and subsequent estimation of individual fluorophore concentrations using a trained machine learning model. The handheld enclosure and internal supports were fabricated using 3D-printed ABS to house the electrical, optical, and thermal subsystems. Power is supplied by a 7.4 V, 2200 mAh lithium-ion battery, which provides power to the motherboard, resistive heating element, and Arduino-based MCU.

The thermal module consists of a resistive heating element (PWR263S-20-2R00J, Digi-Key) affixed to the underside of an aluminum heating block using thermal compound (AATA-5G, Arctic Alumina). Temperature feedback is provided by a thermistor (95C0606, Digi-Key) embedded within the core of the heating block. Sustained temperature regulation is achieved through negative feedback control using an N-channel MOSFET (63J7707, Digi-Key).

The optical module incorporates a blue surface-mount LED (1497-1138-1, Digi-Key; peak emission ~480 nm) for fluorescence excitation and a wide-spectrum integrated color sensor (AS7341, Digi-Key) for optical detection. LED intensity is manually adjustable using a multi-turn cermet trimmer (3296W, Digi-Key). An I^2^C multiplexer (TCA9548A, Digi-Key) enables parallel addressing of multiple optical sensors across the four assay chambers. Excitation light enters the heating block from below, illuminating the assay tube. Fluorescence emission is detected by optical sensors positioned orthogonally (90° off-axis) to the excitation path to minimize excitation light bleed-through. The MCU coordinates excitation timing, sensor readout, heating control, and status indication.

## Survey information

A significant majority of our survey participants were male (60%), with a minority population of female and other (transgender male, transgender female, non-binary, other). We found that most of our participants were between 25 and 34, with an average income of $50,000- $ 99,000. Our results showed that, for the majority of participants, the highest educational level was a bachelor’s degree. The most common environment for respondents was an urban setting, and our surveyed population was primarily white, with a small but present representation of African American and Hispanic populations.

## Neural-network model for fluorophore concentration prediction

A feedforward neural network was used to predict FAM and HEX concentrations from the 8-channel fluorescence measurements acquired by the spectral sensor. The model was formulated as a multivariate regression problem, with the 8-channel fluorescence vector as input and the predicted FAM and HEX concentrations as output. The dataset was divided into training and test subsets using an 80/20 split.

The network comprised an input layer with 8 nodes, hidden layers of size 16, 32, 64, 64, 64, 32, 16, and 8, and an output layer with 2 nodes. ReLU activation was used in the hidden layers, and the output layer was linear. Training was performed using mean squared error loss and the Adam optimizer for 1200 epochs with a batch size of 12.

Model performance was assessed on the held-out test set using the coefficient of determination (R²), mean absolute error (MAE), and mean squared error (MSE). Actual-versus-predicted concentration plots for FAM and HEX were generated from the saved prediction outputs. Residual distributions and agreement analyses were also used to examine prediction error across the tested concentration range.

No data augmentation, hyperparameter tuning, or k-fold cross-validation was performed. The model was trained only on experimentally measured dye-mixture data.

# Supplementary figures

**Figure S1. Software and smartphone app.** a) Block diagram illustrating the interconnections among the components of the portable analyzer. Output includes a resistive heater, an excitation LED, and a status LED bar. Inputs include temperature feedback from the thermistor and a wide-spectrum color sensor. An Arduino Nano controls all modules and interfaces with an Android phone through Bluetooth. b) Block diagram demonstrating the workflow of the Android smartphone app. The GUI will walk patients through the entire testing workflow, confirm each step has been completed, and display results on the last page, with an option to upload them to cloud storage sites.


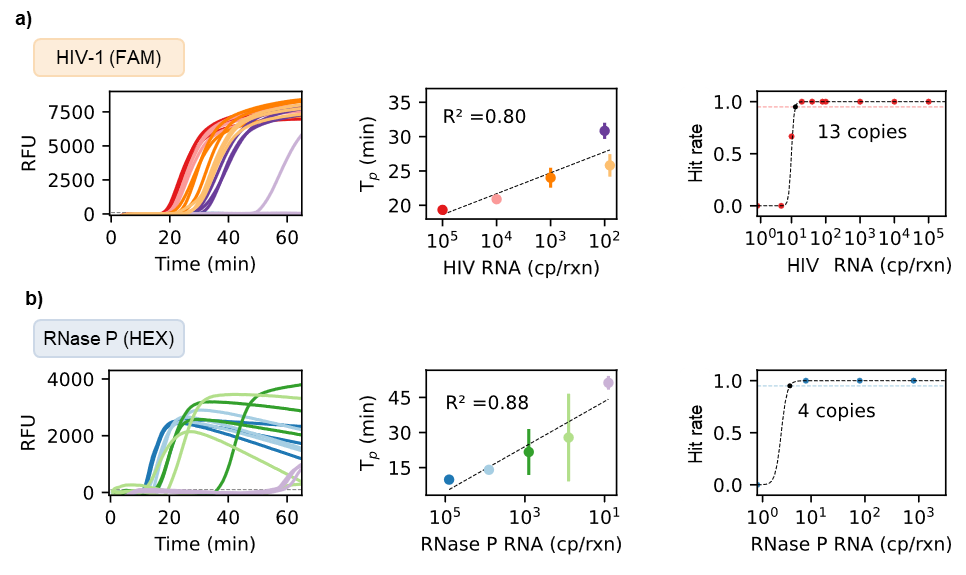


**Figure S2. Single-plex assay validation.** a) The probe-based HIV-1 assay was initially screened using 10^5^ to 1 copy of viral RNA (left). The summary of detection linearity showed an R^2^ of 0.80 (middle), resulting in a limit of detection of 13 copies at 95% confidence level (right). b) Similarly, the probe-based RNase P assay was screened using 10^5^ to 1 copy of RNA (left). The summary of detection linearity showed an R^2^ of 0.88 (middle), resulting in a limit of detection of 4 copies at 95% confidence level (right).


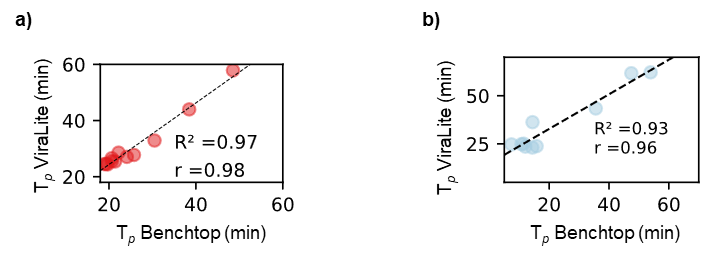


**Figure S3. Correlation between ViraLite portable and benchtop instruments for single-plex RT-LAMP assays.** a) HIV-1 assay showed very strong correlation (r = 0.98) between the two analyzers and great linearity (R^2^ = 0.97) for semi-quantitative analysis. b) RNase P assay also showed very strong correlation (r = 0.96) between analyzers and great linearity (R^2^ = 0.93), showcasing the potential for multiplex detection between both assays, regardless of sensitivity losses.

**
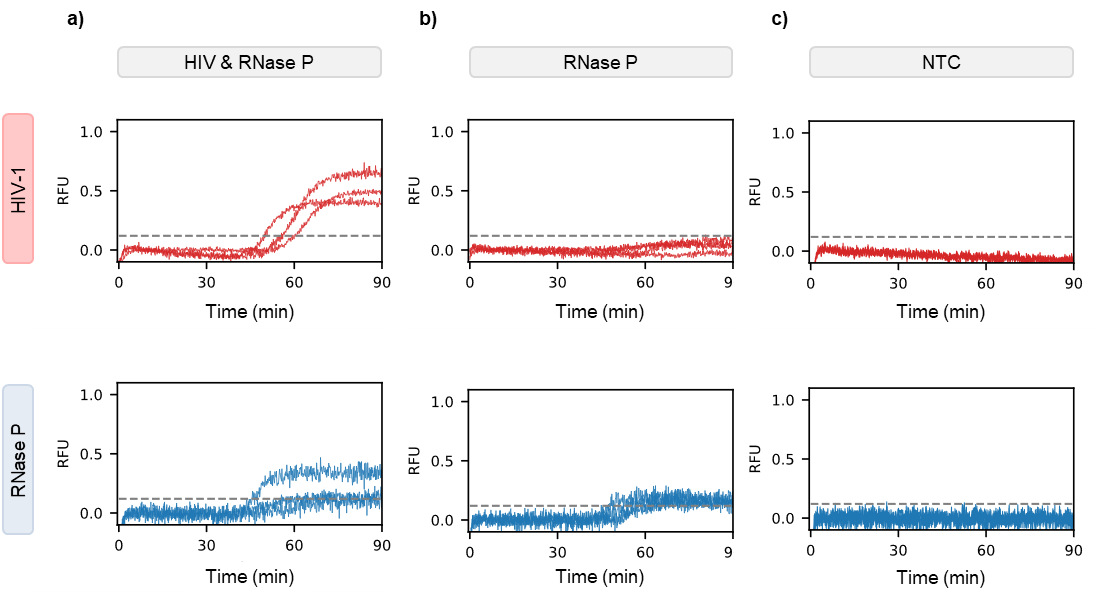
**

**Figure S4. Multiplex cross-reactivity using our portable analyzer.** a) The multiplex assay used FAM and HEX fluorophores to distinguish HIV-1 (top) and RNase P (bottom), respectively. When both targets are present in the sample, exponential amplification occurs for both FAM and HEX. b) For samples with only RNase P, there is only amplification detected in the HEX channel. c) No-Template Control (NTC) shows no amplification for either FAM or HEX (HIV-1 or RNase P).

**
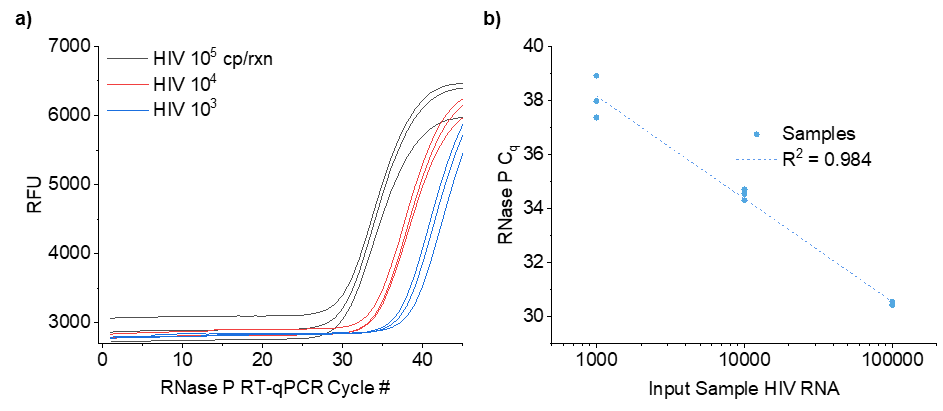
**

**Figure S5. RNase P Validation.** a) RT-qPCR amplification curves from HIV Seracare samples at three diluted concentrations, showing RNase P presence. b) PCR C_q_ showed a linear relationship against input concentration.

**Figure S6. Response of individual sensor channels to increasing dye concentration.** a) Optical sensor readout from five channels showing the crowded yet accurate detection of increasing FAM dye. b) Readout from the same five channels for increasing concentrations of HEX dye. When used together, the two dyes are indistinguishable because both the 515 nm-centered (FAM best) and the 555 nm-centered (HEX best) channels are sensitive to the other dye.


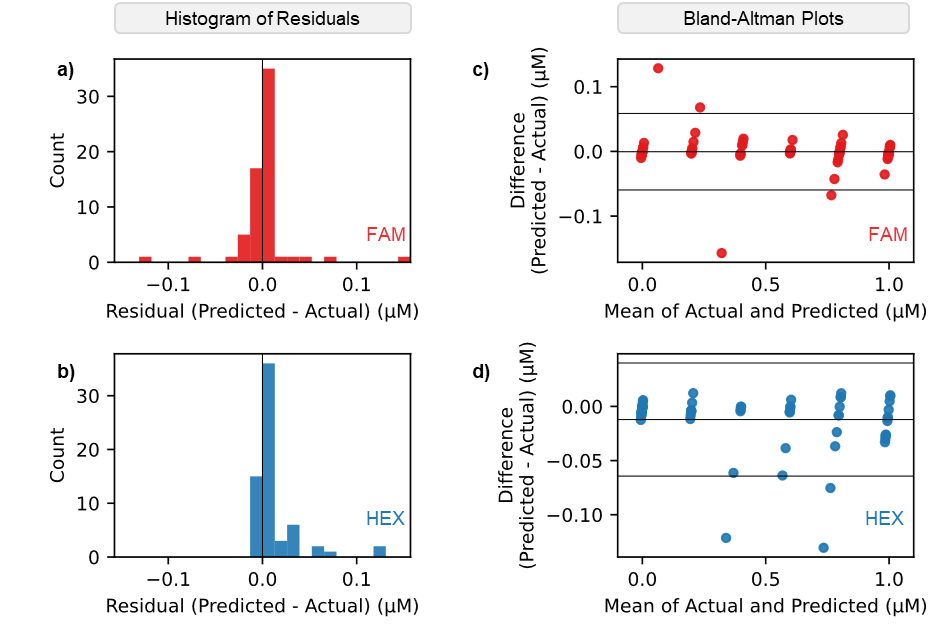


**Figure S7. Residual error distribution and agreement analysis for NN-predicted fluorescence values.** a) and b) Residual histograms for the FAM and HEX channels, respectively, show the distribution of prediction errors, defined as predicted minus reference fluorescence concentration, across all samples. In both channels, residuals are centered near zero with a narrow spread, indicating low bias and limited dispersion of errors. c) and d) Bland–Altman plots for the FAM and HEX channels, respectively, compare predicted and reference fluorescence values by plotting the difference against their mean. The solid horizontal line denotes the mean difference (bias), and dashed lines indicate the limits of agreement (±1.96 SD). The absence of systematic trends and the concentration-independent distribution of residuals demonstrate that NN prediction errors remain stable across the fluorescence range relevant for threshold-based LAMP interpretation.

# Supplementary tables

**Table S1. Multiplex RT-LAMP assay**

| **Component** | **Volume (µL)/rxn** | **Final Concentration** | |
| --- | --- | --- | --- |
| NEB Isothermal Buffer | 5.00 | 1 | X |
| HIV-F3 | 0.38 | 0.15 | µM |
| HIV-B3 | 0.38 | 0.15 | µM |
| HIV-FIP | 0.80 | 0.8 | µM |
| HIV-BIP | 1.20 | 1.2 | µM |
| HIV-LB | 0.60 | 0.6 | µM |
| HIV-LF | 0.60 | 0.6 | µM |
| HIV-QFIP:Fd | 0.40 | 0.4 | µM |
| RNase P-F3 | 0.25 | 0.1 | µM |
| RNase P-B3 | 0.25 | 0.1 | µM |
| RNase P-FIP | 0.80 | 0.8 | µM |
| RNase P-BIP | 0.80 | 0.8 | µM |
| RNase P-LFP:Q | 0.40 | 0.4 | µM |
| RNase P-LB | 0.40 | 0.4 | µM |
| Betaine | 4.00 | 0.4 | M |
| MgSO4 | 3.00 | 6 | mM |
| dNTP Mix | 7.00 | 1.4 | mM |
| Bst2.0 polymerase | 3.44 | 0.55 | U/µl |
| Warmstart Reverse Transcriptase | 1.10 | 0.33 | U/µl |
| RNA | 10.00 | 10 | µl/rxn |

**Table S2. Probe-based RT-LAMP primers**

| **Target** | **Primer** | | **Sequence (5' -> 3')** | **GenBank Target** |
| --- | --- | --- | --- | --- |
| RNase P | F3 | TTGATGAGCTGGAGCCA | | U94316.1 |
| RNase P | B3 | CACCCTCAATGCAGAGTC | | U94316.1 |
| RNase P | FIP | GTGTGACCCTGAAGACTCGGTTTTAGCCACTGACTCGGATC | | U94316.1 |
| RNase P | BIP | CCTCCGTGATATGGCTCTTCGTTTTTTTCTTACATGGCTCTGGTC | | U94316.1 |
| RNase P | LFP | /5HEX/ATGTGGATGGCTGAGTTGTT | | U94316.1 |
| RNase P | LB | CATGCTGAGTACTGGACCTC | | U94316.1 |
| RNase P | Q | CAGCCATCCACAT/3BHQ_1/ | | U94316.1 |
| HIV-1 | F3 | AGTTCCCTTAGATAAAGACTT | | AF033819.3 |
| HIV-1 | B3 | CCTACATACAAATCATCCATGT | | AF033819.3 |
| HIV-1 | FIP | GTGGAAGCACATTGTACTGATATCTTTTTGGAAGTATACTGCATTTACCAT | | AF033819.3 |
| HIV-1 | BIP | GGAAAGGATCACCAGCAATATTCCTCTGGATTTTGTTTTCTAAAAGGC | | AF033819.3 |
| HIV-1 | LB | GCATGACAAAAATCTTAGA | | AF033819.3 |
| HIV-1 | LF | GGTGTCTCATTGTTTATACTA | | AF033819.3 |
| HIV-1 | QFIP | /5IABkFQ/GTGGAAGCACATTGTACTGATATCTTTTTGGAAGTATACTGCATTTACCAT | | AF033819.3 |
| HIV-1 | Fd | GATATCAGTACAATGTGCTTCCAC /36-FAM/ | | AF033819.3 |

**Table S3. One-step RT-qPCR assay**

| **Component** | **Volume (µL)/rxn** | **Final Concentration** | |
| --- | --- | --- | --- |
| Fast Taq One-Step Master Mix (Applied Biosystems) | 6.25 | 1 | X |
| Forward Primer (IDT) | 1.50 | 1.2 | µM |
| Reverse Primer (IDT) | 1.50 | 1.2 | µM |
| Probe (IDT) | 0.63 | 0.5 | µM |
| RNA | 10.00 | variable |  |
| UltraPure Water | 5.13 | - |  |

**Table S4. RT-qPCR temperature cycling**

| **Step** | **Temperature (°C)** | **Time** | **Cycles** |
| --- | --- | --- | --- |
| Reverse transcription | 50 | 5 min | 1 |
| RT inactivation / initial denaturation | 95 | 20 seconds | 1 |
| Denature | 95 | 3 seconds | 40 |
| Anneal / extend | 60 | 30 seconds |  |

**Table S5. RT-qPCR primer and probe sequences**

| **Target** | **Primer** | **Sequence (5' - 3')** |
| --- | --- | --- |
| HIV-1 pol | Forward | CATGTTTTCAGCATTATCAGAAGGA |
| HIV-1 pol | Reverse | TGCTTGATGTCCCCCCACT |
| HIV-1 pol | Probe | FAM-CCACCCCACAAGATTTAAACACCATGCTAA-BHQ1 |

**Table S6. Portable analyzer Bill of Materials**

**Table S7. Clinically archived sample testing by RT-qPCR and multiplexed RT-LAMP**


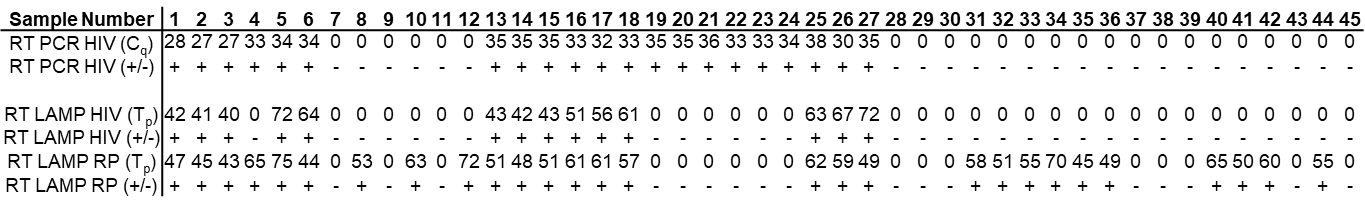


**Table S8. Contextual comparison of ViraLite and the Cepheid Xpert HIV-1 VL platform across workflow, deployment, and selected reported performance characteristics**

| **Parameter** | **ViraLite (this work)** | **Cepheid Xpert HIV-1 VL** |
| --- | --- | --- |
| Intended use | Home/primary-care identification of viral rebound or possible ART failure | Quantitative viral load testing in near-patient, clinic, or laboratory settings |
| Reported sensitivity | 93.30% | 94.50% |
| Reported specificity | 100% | 98.30% |
| Reported LOD | 47 copies/reaction (~ 3.3 x 103 copies/mL of plasma) | 40 copies/mL |
| Time to result | ~75 min | ~90 min |
| Hardware footprint | Two palm-sized modules | Benchtop instrument platform |
| Portability | Portable | Limited, benchtop instrument platform |
| Power requirements | Battery-compatible and plug-in operation | Mains-powered benchtop platform |
| Multiplexing/detection approach | Lens-free fluorescence detection with ML-based unmixing | Fixed cartridge-based molecular detection |
| Adaptability to new targets | High; reconfigurable without optical hardware redesign | Fixed assay architecture; requires a separate cartridge |

**Note:** This table is provided for general reference only and does not represent a direct head-to-head evaluation under matched study conditions.

# Supplementary videos

**Supplementary Video S1. (Duration 2m 57s)** Demonstration of the testing workflow.

**Supplementary Video S2. (Duration 5m 58s)** Initial demonstration of the testing workflow, shown to survey participants before the multiple-choice survey questions.

# References

1. Becherer, L. *et al.* Loop-mediated isothermal amplification (LAMP) – review and classification of methods for sequence-specific detection. *Anal. Methods* **12**, 717–746 (2020).

2. A. Nanayakkara, I. & M. White, I. Demonstration of a quantitative triplex LAMP assay with an improved probe-based readout for the detection of MRSA. *Analyst* **144**, 3878–3885 (2019).

3. Ball, C. S. *et al.* Quenching of unincorporated amplification signal reporters in reverse-transcription loop-mediated isothermal amplification enabling bright, single-step, closed-tube, and multiplexed detection of RNA viruses. *Anal. Chem.* **88**, 3562–3568 (2016).

4. Tanner, N. A., Zhang, Y. & Evans Jr., T. C. Simultaneous multiple target detection in real-time loop-mediated isothermal amplification. *BioTechniques* **53**, 81–89 (2012).

5. Zhang, Y. & Tanner, N. A. Development of multiplexed reverse-transcription loop-mediated isothermal amplification for detection of SARS-CoV-2 and influenza viral RNA. *BioTechniques* **70**, 167–174 (2021).

6. Bhadra, S. *et al.* Real-time sequence-validated loop-mediated isothermal amplification assays for detection of middle east respiratory syndrome coronavirus (MERS-CoV). *PLOS ONE* **10**, e0123126 (2015).

7. Jiang, Y. S. *et al.* Robust strand exchange reactions for the sequence-specific, real-time detection of nucleic acid amplicons. *Anal. Chem.* **87**, 3314–3320 (2015).

8. Kshirsagar, A., Politza, A. J. & Guan, W. Deep learning enabled universal multiplexed fluorescence detection for point-of-care applications. *ACS Sens.* **9**, 4017–4027 (2024).
